# Supplementary material for: Identification of circadian clock modulators from existing drugs
Source: EMBO Mol Med. 2018 Apr 17;10(5):e8724. doi: 10.15252/emmm.201708724 (PMC5938619; doi:10.15252/emmm.201708724)
Supplement: Supplementary file 1 — Appendix [file EMMM-10-e8724-s001.pdf]

Appendix: Identification of circadian clock modulators from existing drugs

Table of Contents:

Appendix Figure S1. Dose-dependent effect of hit compounds.

Appendix Figure S2. Effect of endogenous steroid hormones on circadian period in U2OS cells.

Appendix Figure S3. Effect of siRNA-mediated knockdown on mRNA levels.

Appendix Figure S4. No signal was observed in *in situ* negative controls.

Appendix Figure S5. Media components do not interfere with the effect of hit compounds DHEA, Dasatinib and Nilotinib.

Appendix Table S1. Statistical information for main Figures.

Appendix Table S2. Statistical information for Expanded View and Appendix Figures.

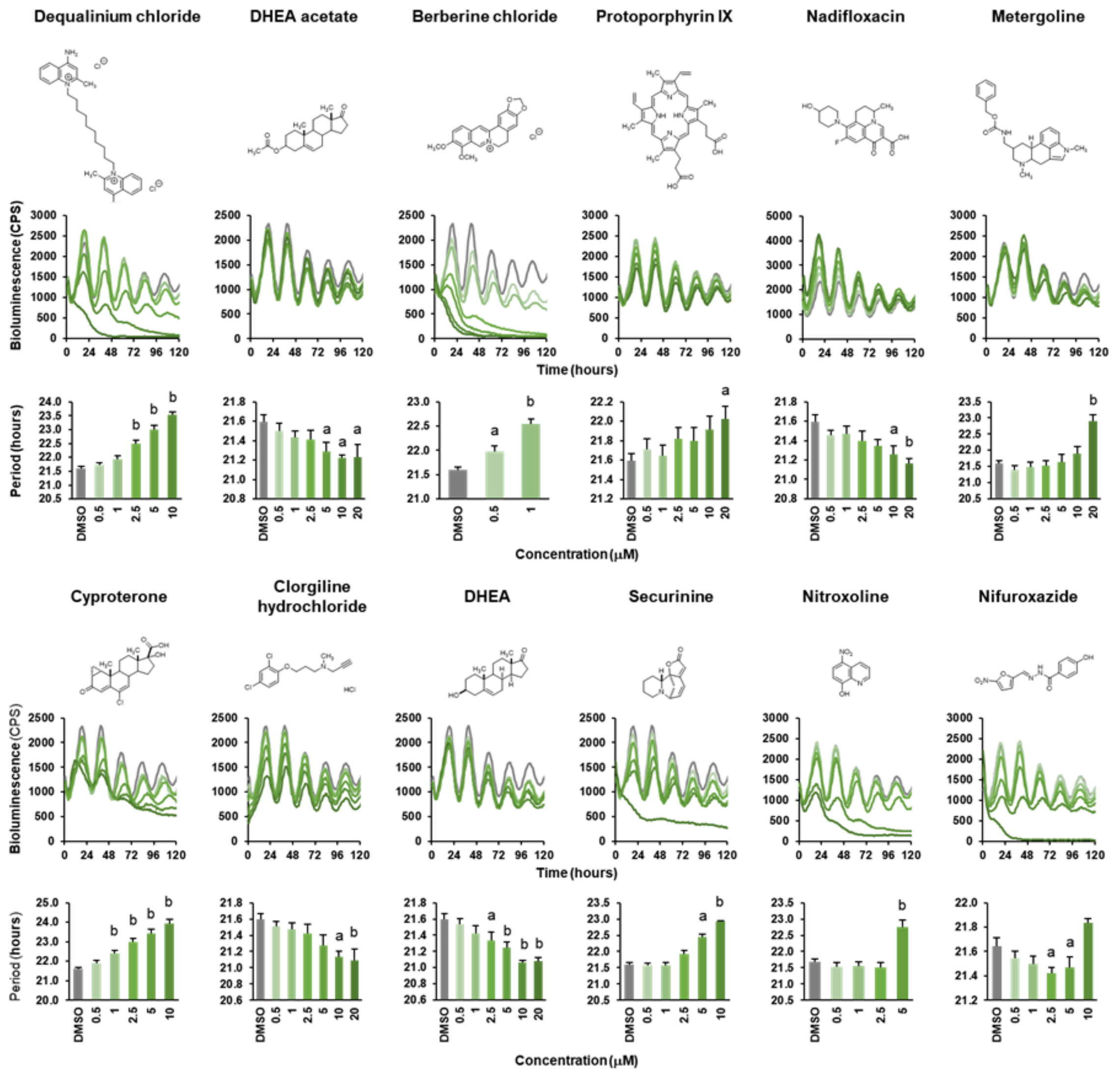

Appendix Figure S1. Dose-dependent effect of hit compounds.

Secondary screening revealed that 59 of 72 potential hit compounds had significant dose-dependent effects on circadian period in U2OS cells. Chemical names and structures are shown for each hit compound above luminescent traces from one of 3 or 4 independent experiments. Histograms below show dose-dependent effects on circadian period, and results are presented as the mean ± SEM of 3 or 4 experiments. Data were analyzed by one-way ANOVA, followed by a Dunnett's test (^{a} p < 0.05, ^{b} p < 0.01). All statistical information is shown in Appendix Table S2.

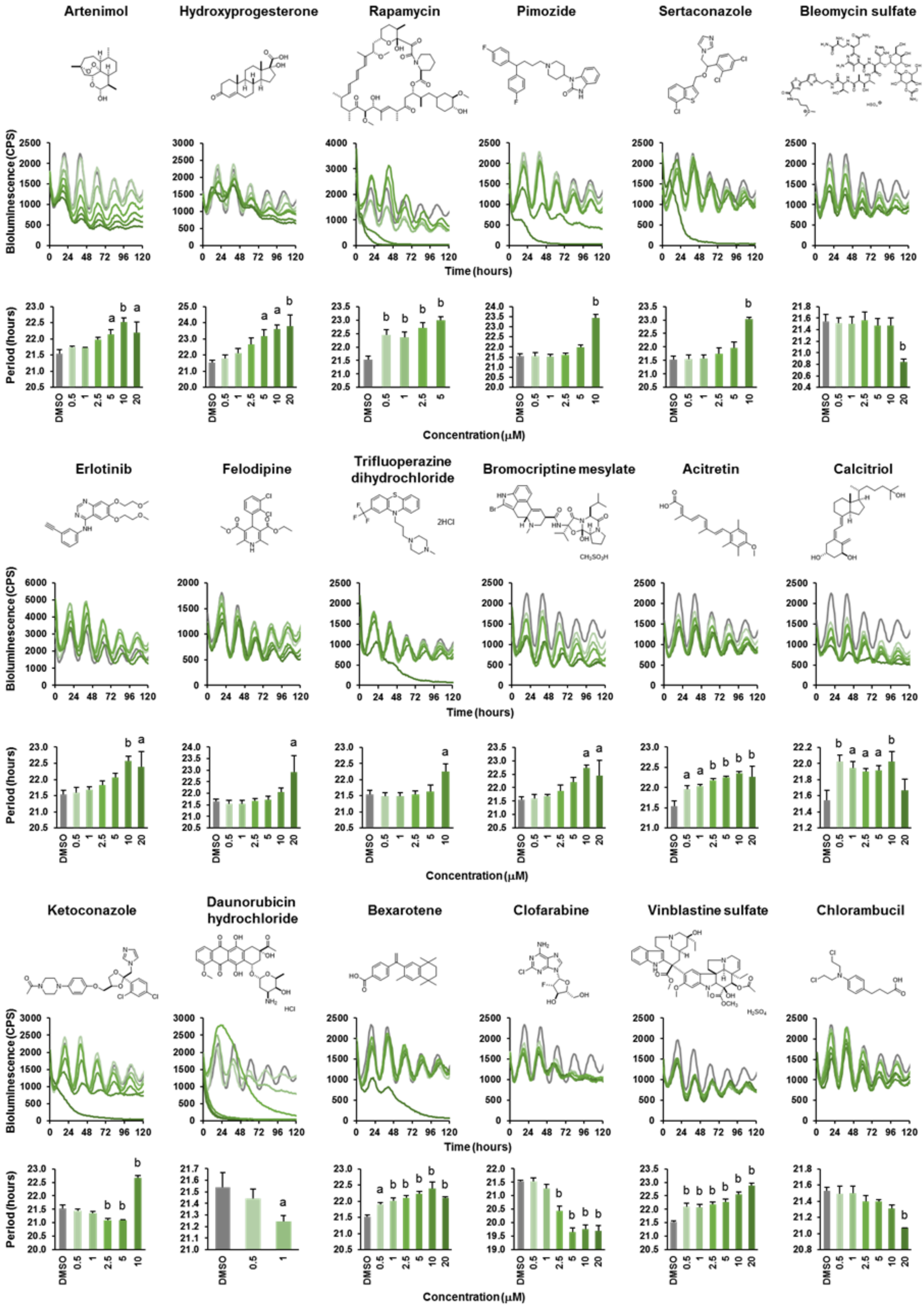

Appendix Figure S1. (continued)

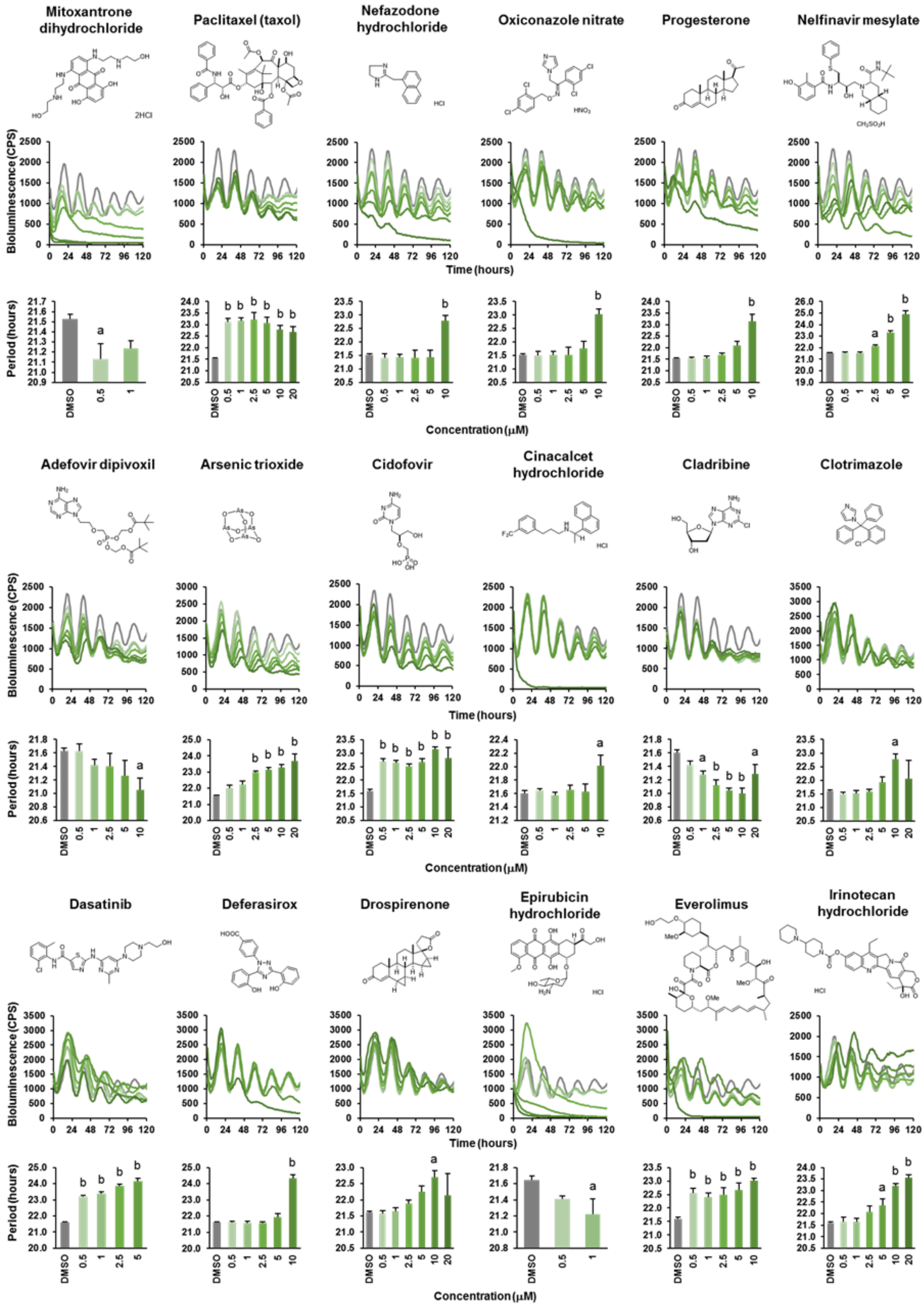

Appendix Figure S1. (continued)

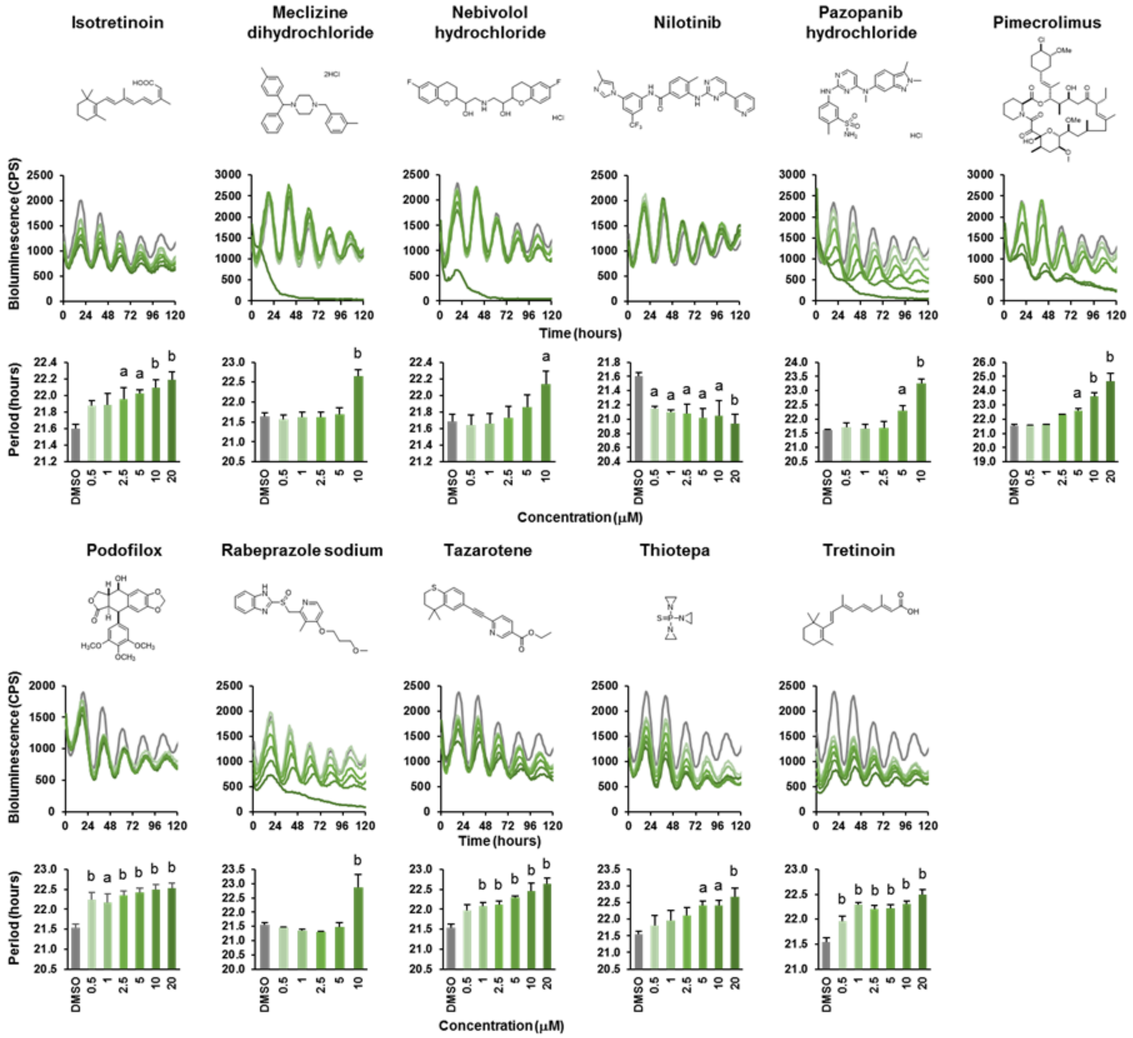

Appendix Figure S1. (continued)

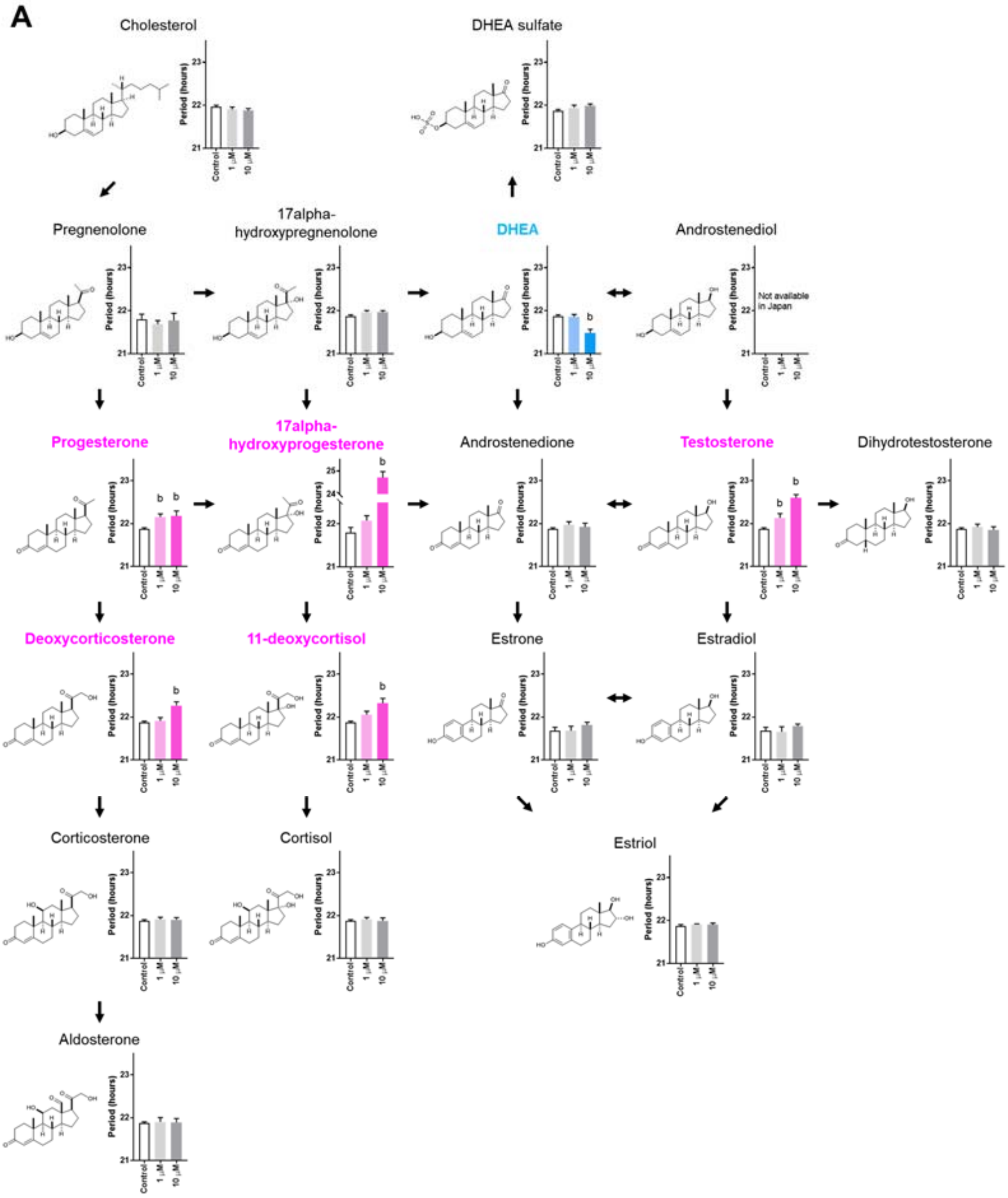

Appendix Figure S2. Effect of endogenous steroid hormones on circadian period in U2OS cells.

A. Biosynthetic pathway and structure of endogenous steroid hormones tested are shown. Histograms show the effects of DMSO control (white bar) and steroids at 1 µM (light color) and 10 µM (darker color) on circadian period in U2OS cells. Compounds that lengthened period are highlighted in pink, and those that shortened circadian period are highlighted in blue. Results are presented as the mean ± SEM. Data were analyzed by one-way ANOVA, followed by a Dunnett's test ([a] $p < 0.05$, [b] $p < 0.01$). All statistical information is shown in Appendix Table S2.

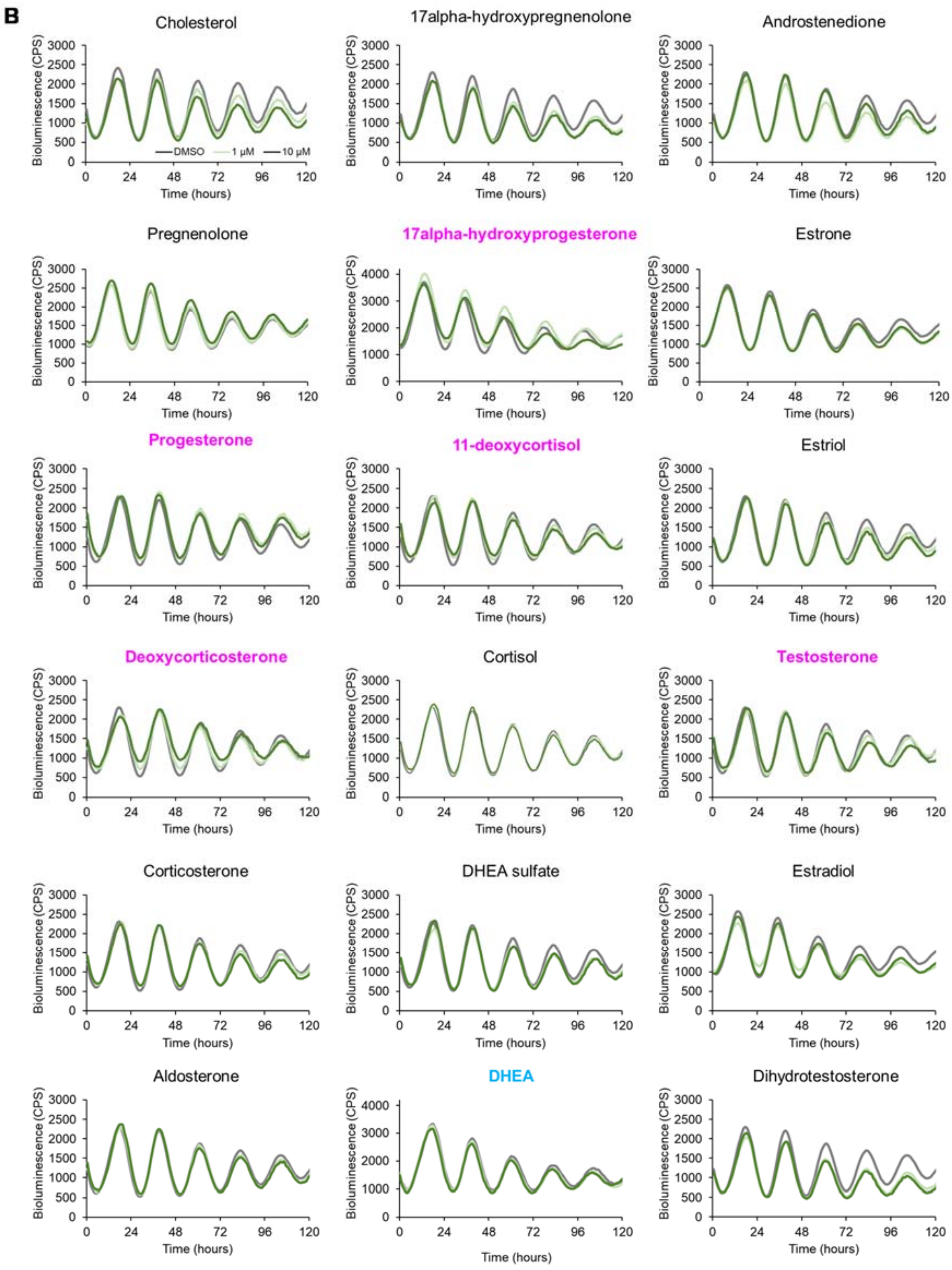

Appendix Figure S2. (continued)

B. Luminescent traces from one experiment is shown and reveals dose-dependent effects of various endogenous steroid hormones tested.

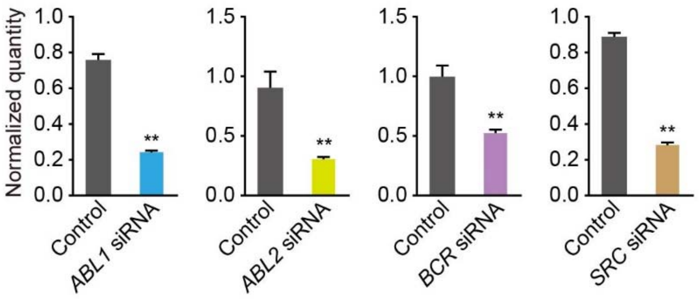

Appendix Figure S3. Effect of siRNA-mediated knockdown on mRNA levels. mRNA levels were analyzed by qPCR and normalized to *GAPDH*. Data are presented as the mean ± SEM of 3 or 4 independent experiments (** $p < 0.01$, Welch's *t*-test). All statistical information is shown in Appendix Table S2.

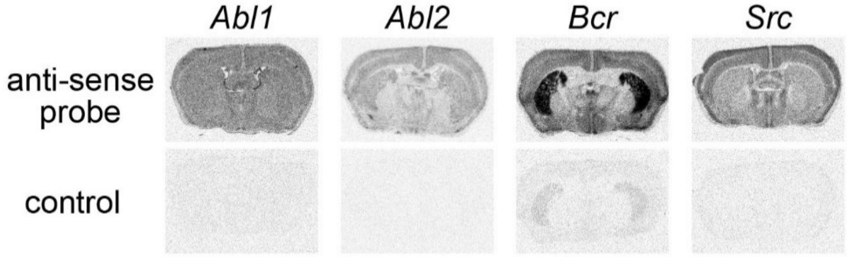

Appendix Figure S4. No signal was observed in *in situ* negative controls.
Representative autoradiograms. Negative control sections were hybridized in the presence of excess unlabelled probe.

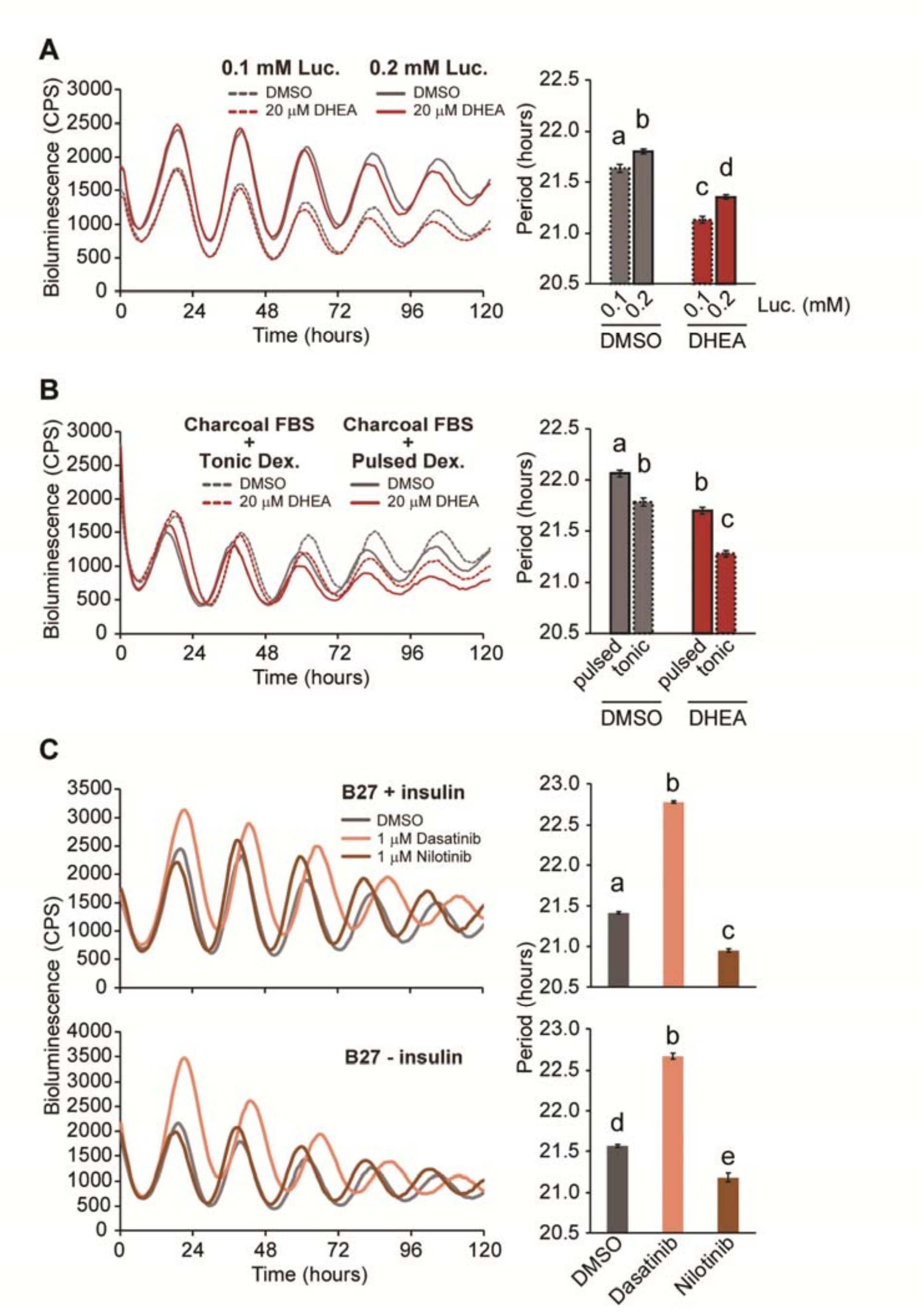

Appendix Figure S5. Media components do not interfere with the effects of hit compounds DHEA, Dasatinib and Nilotinib.

A. DHEA shortens circadian period in the presence of different luciferin (Luc) concentrations in the media (0.1 mM versus 0.2 mM). Results are presented as the mean ± SEM (n = 6). Data were analyzed by two-way ANOVA, followed by a Sidak's multiple comparisons test ($p < 0.01$). All statistical information is shown in Appendix Table S2.

B. DHEA shortens circadian period in the absence of corticosterone or other steroid hormones in the media. Results are presented as the mean ± SEM (n = 6). Data were analyzed by two-way ANOVA, followed by a Sidak's multiple comparisons test ($p < 0.01$). All statistical information is shown in Appendix Table S2.

C. The presence or absence of insulin in the media supplement B27 did not influence the period-lengthening or -shortening effects of tyrosine kinase inhibitors, Dasatinib and Nilotinib, respectively. Results are presented as the mean ± SEM (n = 6). Data were analyzed by two-way ANOVA, followed by a Sidak's multiple comparisons test ($p < 0.05$). All statistical information is shown in Appendix Table S2.

Appendix Table S1: Statistical information for main figures

Figure 2A	Dunnett's multiple comparison test	P value	N	Label
	DMSO		3	
	0.5 vs DMSO	0.4329	3	
	1 vs DMSO	0.1945	3	
	2.5 vs DMSO	0.0380	3	*
	5 vs DMSO	0.0042	3	**
	10 vs DMSO	<0.0001	3	**
	20 vs DMSO	<0.0001	3	**

Figure 2B	Welch's t-test	P value (two-tailed)	N	Label
MEF	DMSO		5	
	DHEA vs DMSO	0.0040	4	**
SCN	DMSO		4	
	DHEA vs DMSO	0.0397	4	*
Lung	DMSO		4	
	DHEA vs DMSO	0.0247	5	*

Figure 2C	two-way ANOVA	P value	N	Label
activity onset	Interaction	0.012		*
	Row Factor	0.0002		**
	Column Factor	<0.0001		**
	Sidak's multiple comparisons test	P value (two-tailed)	N	Label
	Control		8	
	DHEA 0% vs Control	0.8985	14	
	DHEA 0.5% vs Control	0.038	14	*
	DHEA 1% vs Control	<0.0001	14	**
	DHEA 0% vs Control	0.754	14	
activity offset	two-way ANOVA	P value	N	Label
	Interaction	0.001		**
	Row Factor	<0.0001		**
	Column Factor	0.0002		**
	Sidak's multiple comparisons test	P value (two-tailed)	N	Label
	DHEA 0% vs Control	>0.9999	14	
	DHEA 0.5% vs Control	0.0006	14	**
	DHEA 1% vs Control	0.0004	14	**
	DHEA 0% vs Control	0.9965	14	

Figure 3A	Welch's t-test	P value (two-tailed)	N	Label
	Normal food		12	
	day1: DHEA vs Normal food	<0.0001	11	**
	day2: DHEA vs Normal food	<0.0001	11	**
	day3: DHEA vs Normal food	<0.0001	11	**
	day4: DHEA vs Normal food	<0.0001	11	**
	day5: DHEA vs Normal food	<0.0001	11	**
	day6: DHEA vs Normal food	<0.0001	11	**
	day7: DHEA vs Normal food	<0.0001	11	**
	day8: DHEA vs Normal food	0.0001	11	**

Figure 3B	Welch's t-test	P value (two-tailed)	N	Label
	Normal food		10	
	day1: DHEA vs Normal food	<0.0001	11	**
	day2: DHEA vs Normal food	0.0001	11	**
	day3: DHEA vs Normal food	0.0215	11	*
	day4: DHEA vs Normal food	0.0017	11	**
	day5: DHEA vs Normal food	0.3024	11	
	day6: DHEA vs Normal food	0.7197	11	
	day7: DHEA vs Normal food	0.8183	11	

Figure 3D	Welch's t-test	P value (two-tailed)	N	Label
	Normal food		10	
	day1: DHEA vs Normal food	0.0150	11	*
	day2: DHEA vs Normal food	0.0013	11	**
	day3: DHEA vs Normal food	0.0101	11	*
	day4: DHEA vs Normal food	0.0580	11	
	day5: DHEA vs Normal food	0.0504	11	
	day6: DHEA vs Normal food	0.0401	11	*
	day7: DHEA vs Normal food	0.0344	11	*
	day8: DHEA vs Normal food	0.0369	11	*

Figure 4A	Dunnet's test	P value (two-tailed)	N	Label
Dasatinib	DMSO		4	
	0.01 vs DMSO	0.9994	3	
	0.05 vs DMSO	0.8198	3	
	0.1 vs DMSO	0.9881	4	
	0.25 vs DMSO	0.3767	4	
	0.5 vs DMSO	0.0035	4	**
	1 vs DMSO	0.0001	4	**
	2.5 vs DMSO	0.0001	4	**
	5 vs DMSO	0.0001	4	**
Nilotinib	DMSO		4	
	0.01 vs DMSO	0.9860	3	
	0.05 vs DMSO	0.0579	3	
	0.1 vs DMSO	0.0013	4	**
	0.25 vs DMSO	0.0001	4	**
	0.5 vs DMSO	0.0001	4	**
	1 vs DMSO	0.0001	4	**
	2.5 vs DMSO	0.0005	4	**
	5 vs DMSO	0.0001	4	**
	10 vs DMSO	0.0003	4	**
	20 vs DMSO	0.0001	4	**

Figure 4B	Welch's t-test	P value (two-tailed)	N	Label
	Control		15	
	ABL1 vs Control	<0.0001	9	**
	Control		15	
	ABL2 vs Control	0.0001	9	**
	Control		15	
	BCR vs Control	0.0151	6	*
	Control		15	
	SRC vs Control	0.1123	6	

Figure 4C	least squares regression with sinusoids	R square	N	Label
Abl1		0.2286		
	ZT2		3	
	ZT6		3	
	ZT10		3	
	ZT14		3	
	ZT18		3	
	ZT22		3	
Abl2		Not converged		
	ZT2		3	
	ZT6		3	
	ZT10		3	
	ZT14		3	
	ZT18		3	
	ZT22		3	

Bcr		0.5316		
	ZT2		3	
	ZT6		3	
	ZT10		3	
	ZT14		3	
	ZT18		3	
	ZT22		3	
Src		0.0498		
	ZT2		3	
	ZT6		3	
	ZT10		3	
	ZT14		3	
	ZT18		3	
	ZT22		3	

Appendix Table S2: Statistical information for Expanded View and Appendix figures

Figure EV1	Dunnett's multiple comparison test	P value	N	Label
	DMSO		6	
	20 vs DMSO	0.0001	6	**
	50 vs DMSO	0.001	6	**
	100 vs DMSO	0.0001	6	**

Figure EV2	two-way ANOVA	P value	N	Label
	Interaction	0.0005		**
	Row Factor	0.0001		**
	Column Factor	<0.0001		**
	Sidak's multiple comparisons test	P value (two-tailed)	N	Label
	Normal food		8	
	DHEA 0% vs Normal food 0%	>0.9999	13	
	DHEA 0.5% vs Normal food 5%	<0.0001	13	**
	DHEA 1% vs Normal food 1%	0.0011	13	**
	DHEA 0% vs Normal food 0%	0.9996	13	

Figure EV5	two-way ANOVA	P value	N	Label
Imatinib	DMSO		4	
	0.01 vs DMSO	0.9710	3	
	0.05 vs DMSO	0.9997	3	
	0.1 vs DMSO	0.9691	4	
	0.25 vs DMSO	0.8616	4	
	0.5 vs DMSO	0.3546	4	
	1 vs DMSO	0.0275	4	*
	2.5 vs DMSO	0.0018	4	**
	5 vs DMSO	0.0007	4	**
	10 vs DMSO	0.0018	4	**
	20 vs DMSO	0.0449	4	*
Bafetinib	DMSO		4	
	0.01 vs DMSO	0.9999	3	
	0.05 vs DMSO	0.2647	3	
	0.1 vs DMSO	0.0016	4	**
	0.25 vs DMSO	0.0001	4	**
	0.5 vs DMSO	0.0001	4	**
	1 vs DMSO	0.0001	4	**
	2.5 vs DMSO	0.0001	4	**
	5 vs DMSO	0.0001	4	**
	10 vs DMSO	0.0555	4	
Bosutinib	DMSO		4	
	0.01 vs DMSO	0.9999	3	
	0.05 vs DMSO	0.9267	3	
	0.1 vs DMSO	0.4436	4	
	0.25 vs DMSO	0.3157	4	
	0.5 vs DMSO	0.2791	4	
	1 vs DMSO	0.6984	4	
	2.5 vs DMSO	0.7495	4	
	5 vs DMSO	0.9997	4	
	10 vs DMSO	0.0008	4	**
Ponatinib	DMSO		4	
	0.01 vs DMSO	0.02	3	*
	0.05 vs DMSO	0.0038	3	**
	0.1 vs DMSO	0.0003	4	**
	0.25 vs DMSO	0.003	4	**
	0.5 vs DMSO	0.9994	4	
	1 vs DMSO	0.0037	4	**
	2.5 vs DMSO	0.0001	4	**
	5 vs DMSO	0.0001	4	**

Appendix Figure S1	Dunnett's multiple comparison test	P value	N	Label
Desqualium chloride	DMSO		3	
	0.5 vs DMSO	0.5064	3	
	1 vs DMSO	0.0863	3	
	2.5 vs DMSO	0.0002	3	b
	5 vs DMSO	<0.0001	3	b
	10 vs DMSO	<0.0001	3	b
DHEA acetate	DMSO		3	
	0.5 vs DMSO	0.5522	3	
	1 vs DMSO	0.3109	3	
	2.5 vs DMSO	0.2449	3	
	5 vs DMSO	0.0481	3	a
	10 vs DMSO	0.0173	3	a
	20 vs DMSO	0.0206	3	a
Berberine chloride	DMSO		3	
	0.5 vs DMSO	0.0322	3	a
	1 vs DMSO	0.0005	3	b
Protoporphyrin IX	DMSO		3	
	0.5 vs DMSO	0.5549	3	
	1 vs DMSO	0.7480	3	
	2.5 vs DMSO	0.2441	3	
	5 vs DMSO	0.2967	3	
	10 vs DMSO	0.0987	3	
	20 vs DMSO	0.0263	3	a
Nadifloxacin	DMSO		3	
	0.5 vs DMSO	0.3252	3	
	1 vs DMSO	0.3674	3	
	2.5 vs DMSO	0.1592	3	
	5 vs DMSO	0.0680	3	
	10 vs DMSO	0.0148	3	a
	20 vs DMSO	0.0030	3	b
Metergoline	DMSO		3	
	0.5 vs DMSO	0.9782	3	
	1 vs DMSO	0.9503	3	
	2.5 vs DMSO	0.9236	3	
	5 vs DMSO	0.8088	3	
	10 vs DMSO	0.3337	3	
	20 vs DMSO	0.0002	3	b
Cyproterone	DMSO		3	
	0.5 vs DMSO	0.0591	3	
	1 vs DMSO	0.0001	3	b
	2.5 vs DMSO	<0.0001	3	b
	5 vs DMSO	<0.0001	3	b
	10 vs DMSO	<0.0001	3	b
Clorgiline hydrochloride	DMSO		3	
	0.5 vs DMSO	0.6360	3	
	1 vs DMSO	0.5266	3	
	2.5 vs DMSO	0.3497	3	
	5 vs DMSO	0.0756	3	
	10 vs DMSO	0.0112	3	a
	20 vs DMSO	0.0061	3	b
DHEA	DMSO		3	
	0.5 vs DMSO	0.6365	3	
	1 vs DMSO	0.1946	3	
	2.5 vs DMSO	0.0465	3	a
	5 vs DMSO	0.0091	3	b
	10 vs DMSO	0.0003	3	b
	20 vs DMSO	0.0004	3	b
Securinine	DMSO		3	
	0.5 vs DMSO	0.8622	3	
	1 vs DMSO	0.8513	3	
	2.5 vs DMSO	0.3166	3	
	5 vs DMSO	0.0142	3	a
	10 vs DMSO	0.0006	3	b

Nitroxoline	DMSO		3	
	0.5 vs DMSO	0.9516	4	
	1 vs DMSO	0.9364	4	
	2.5 vs DMSO	0.9637	4	
	5 vs DMSO	0.0002	4	b
Nifuroxazide	DMSO		3	
	0.5 vs DMSO	0.3315	4	
	1 vs DMSO	0.1636	4	
	2.5 vs DMSO	0.0318	4	a
	5 vs DMSO	0.0472	4	a
	10 vs DMSO	0.9997	4	
Artenimol	DMSO		3	
	0.5 vs DMSO	0.4996	3	
	1 vs DMSO	0.5235	3	
	2.5 vs DMSO	0.1126	3	
	5 vs DMSO	0.0316	3	a
	10 vs DMSO	0.0010	3	b
	20 vs DMSO	0.0176	3	a
Hydroxyprogesterone	DMSO		3	
	0.5 vs DMSO	0.7031	3	
	1 vs DMSO	0.4511	3	
	2.5 vs DMSO	0.1287	3	
	5 vs DMSO	0.0248	3	a
	10 vs DMSO	0.0127	3	a
	20 vs DMSO	0.0033	3	b
Rapamycin	DMSO		3	
	0.5 vs DMSO	0.0042	3	b
	1 vs DMSO	0.0077	3	b
	2.5 vs DMSO	0.0007	3	b
	5 vs DMSO	0.0001	3	b
Pimozide	DMSO		3	
	0.5 vs DMSO	0.8085	3	
	1 vs DMSO	0.8608	3	
	2.5 vs DMSO	0.7223	3	
	5 vs DMSO	0.0564	3	
	10 vs DMSO	<0.0001	3	b
Sertaconazole	DMSO		3	
	0.5 vs DMSO	0.8123	3	
	1 vs DMSO	0.7957	3	
	2.5 vs DMSO	0.4284	3	
	5 vs DMSO	0.1291	3	
	10 vs DMSO	<0.0001	3	b
Bleomycin sulfate	DMSO		3	
	0.5 vs DMSO	0.7987	3	
	1 vs DMSO	0.7735	3	
	2.5 vs DMSO	0.8979	3	
	5 vs DMSO	0.6882	3	
	10 vs DMSO	0.6882	3	
	20 vs DMSO	0.0011	3	b
Erlotinib	DMSO		3	
	0.5 vs DMSO	0.7970	3	
	1 vs DMSO	0.6835	3	
	2.5 vs DMSO	0.4665	3	
	5 vs DMSO	0.1879	3	
	10 vs DMSO	0.0088	3	b
	20 vs DMSO	0.0288	3	a
Felodipine	DMSO		3	
	0.5 vs DMSO	0.9028	4	
	1 vs DMSO	0.9028	4	
	2.5 vs DMSO	0.8335	4	
	5 vs DMSO	0.8024	4	
	10 vs DMSO	0.4501	4	
	20 vs DMSO	0.0154	4	a

Trifluoperazine dihydrochloride	DMSO		3	
	0.5 vs DMSO	0.8952	3	
	1 vs DMSO	0.8952	3	
	2.5 vs DMSO	0.8279	3	
	5 vs DMSO	0.6954	3	
	10 vs DMSO	0.0102	3	a
Bromocriptine mesylate	DMSO		3	
	0.5 vs DMSO	0.8065	3	
	1 vs DMSO	0.7256	3	
	2.5 vs DMSO	0.4582	3	
	5 vs DMSO	0.1395	3	
	10 vs DMSO	0.0103	3	a
	20 vs DMSO	0.0427	3	a
Acitretin	DMSO		3	
	0.5 vs DMSO	0.0359	3	a
	1 vs DMSO	0.0163	3	a
	2.5 vs DMSO	0.0028	3	b
	5 vs DMSO	0.0013	3	b
	10 vs DMSO	0.0003	3	b
	20 vs DMSO	0.0010	3	b
Calcitriol	DMSO		3	
	0.5 vs DMSO	0.0037	3	b
	1 vs DMSO	0.0121	3	a
	2.5 vs DMSO	0.0238	3	a
	5 vs DMSO	0.0201	3	a
	10 vs DMSO	0.0037	3	b
	20 vs DMSO	0.4943	3	
Ketoconazole	DMSO		3	
	0.5 vs DMSO	0.7208	3	
	1 vs DMSO	0.2308	3	
	2.5 vs DMSO	0.0027	3	b
	5 vs DMSO	0.0032	3	b
	10 vs DMSO	<0.0001	3	b
Daunorubicin hydrocholoride	DMSO		3	
	0.5 vs DMSO	0.2588	3	
	1 vs DMSO	0.0149	3	a
Bexarotene	DMSO		3	
	0.5 vs DMSO	0.0364	3	a
	1 vs DMSO	0.0100	3	b
	2.5 vs DMSO	0.0027	3	b
	5 vs DMSO	0.0006	3	b
	10 vs DMSO	<0.0001	3	b
	20 vs DMSO	0.0069	3	b
Clofarabine	DMSO		3	
	0.5 vs DMSO	0.8662	3	
	1 vs DMSO	0.3588	3	
	2.5 vs DMSO	0.0004	3	b
	5 vs DMSO	<0.0001	3	b
	10 vs DMSO	<0.0001	3	b
	20 vs DMSO	<0.0001	3	b
Vinblastine sulfate	DMSO		3	
	0.5 vs DMSO	0.0024	3	b
	1 vs DMSO	0.0032	3	b
	2.5 vs DMSO	0.0006	3	b
	5 vs DMSO	0.0002	3	b
	10 vs DMSO	<0.0001	3	b
	20 vs DMSO	<0.0001	3	b
Chlorambucil	DMSO		3	
	0.5 vs DMSO	0.7081	3	
	1 vs DMSO	0.7590	3	
	2.5 vs DMSO	0.2627	3	
	5 vs DMSO	0.2627	3	
	10 vs DMSO	0.0507	3	
	20 vs DMSO	0.0002	3	b

Mitoxantrone dihydrochloride	DMSO		3	
	0.5 vs DMSO	0.0292	3	a
	1 vs DMSO	0.0736	3	
Paclitaxel (taxol)	DMSO		3	
	0.5 vs DMSO	0.0002	3	b
	1 vs DMSO	0.0001	3	b
	2.5 vs DMSO	0.0001	3	b
	5 vs DMSO	0.0003	3	b
	10 vs DMSO	0.0017	3	b
	20 vs DMSO	0.0032	3	b
Nefazodone hydrochloride	DMSO		3	
	0.5 vs DMSO	0.9626	3	
	1 vs DMSO	0.9559	3	
	2.5 vs DMSO	0.9626	3	
	5 vs DMSO	0.9483	3	
	10 vs DMSO	<0.0001	3	b
Oxiconazole nitrate	DMSO		3	
	0.5 vs DMSO	0.8745	3	
	1 vs DMSO	0.8399	3	
	2.5 vs DMSO	0.8579	3	
	5 vs DMSO	0.2589	3	
	10 vs DMSO	<0.0001	3	b
Progesterone	DMSO		3	
	0.5 vs DMSO	0.8564	3	
	1 vs DMSO	0.8564	3	
	2.5 vs DMSO	0.6397	3	
	5 vs DMSO	0.1285	3	
	10 vs DMSO	0.0002	3	b
Nelfinavir mesylate	DMSO		3	
	0.5 vs DMSO	0.7851	3	
	1 vs DMSO	0.8042	3	
	2.5 vs DMSO	0.0139	3	a
	5 vs DMSO	<0.0001	3	b
	10 vs DMSO	<0.0001	3	b
Adefovir dipivoxil	DMSO		3	
	0.5 vs DMSO	0.8402	4	
	1 vs DMSO	0.3673	4	
	2.5 vs DMSO	0.3310	4	
	5 vs DMSO	0.1187	4	
	10 vs DMSO	0.0145	4	a
Arsenic trioxide	DMSO		3	
	0.5 vs DMSO	0.2215	3	
	1 vs DMSO	0.0790	3	
	2.5 vs DMSO	0.0007	3	b
	5 vs DMSO	0.0003	3	b
	10 vs DMSO	0.0001	3	b
	20 vs DMSO	<0.0001	3	b
Cidofovir	DMSO		3	
	0.5 vs DMSO	0.0013	3	b
	1 vs DMSO	0.0020	3	b
	2.5 vs DMSO	0.0053	3	b
	5 vs DMSO	0.0016	3	b
	10 vs DMSO	<0.0001	3	b
	20 vs DMSO	0.0005	3	b
Cinacalcet hydrochloride	DMSO		3	
	0.5 vs DMSO	0.7649	3	
	1 vs DMSO	0.8939	3	
	2.5 vs DMSO	0.7371	3	
	5 vs DMSO	0.7910	3	
	10 vs DMSO	0.0357	3	a

Cladribine	DMSO		3	
	0.5 vs DMSO	0.2250	3	
	1 vs DMSO	0.0263	3	a
	2.5 vs DMSO	0.0019	3	b
	5 vs DMSO	0.0005	3	b
	10 vs DMSO	0.0003	3	b
	20 vs DMSO	0.0315	3	a
Clotrimazole	DMSO		3	
	0.5 vs DMSO	0.9190	3	
	1 vs DMSO	0.9088	3	
	2.5 vs DMSO	0.8720	3	
	5 vs DMSO	0.5458	3	
	10 vs DMSO	0.0234	3	a
	20 vs DMSO	0.3958	3	
Dasatinib	DMSO		3	
	0.5 vs DMSO	<0.0001	3	b
	1 vs DMSO	<0.0001	3	b
	2.5 vs DMSO	<0.0001	3	b
	5 vs DMSO	<0.0001	3	b
Deferasirox	DMSO		3	
	0.5 vs DMSO	0.8340	3	
	1 vs DMSO	0.8649	3	
	2.5 vs DMSO	0.8742	3	
	5 vs DMSO	0.3547	3	
	10 vs DMSO	<0.0001	3	b
Drospirenone	DMSO		3	
	0.5 vs DMSO	0.8709	3	
	1 vs DMSO	0.8282	3	
	2.5 vs DMSO	0.6088	3	
	5 vs DMSO	0.2596	3	
	10 vs DMSO	0.0490	3	a
	20 vs DMSO	0.3566	3	
Epirubicin hydrochloride	DMSO		3	
	0.5 vs DMSO	0.1549	4	
	1 vs DMSO	0.0285	4	a
Everolimus	DMSO		3	
	0.5 vs DMSO	0.0010	3	b
	1 vs DMSO	0.0034	3	b
	2.5 vs DMSO	0.0014	3	b
	5 vs DMSO	0.0004	3	b
	10 vs DMSO	<0.0001	3	b
Irinotecan hydrochloride	DMSO		3	
	0.5 vs DMSO	0.7899	3	
	1 vs DMSO	0.8049	3	
	2.5 vs DMSO	0.1423	3	
	5 vs DMSO	0.0189	3	a
	10 vs DMSO	<0.0001	3	b
	20 vs DMSO	<0.0001	3	b
Isotretinoin	DMSO		3	
	0.5 vs DMSO	0.1059	3	
	1 vs DMSO	0.0922	3	
	2.5 vs DMSO	0.0383	3	a
	5 vs DMSO	0.0151	3	a
	10 vs DMSO	0.0058	3	b
	20 vs DMSO	0.0014	3	b
Meclizine dihydrochloride	DMSO		3	
	0.5 vs DMSO	0.9352	4	
	1 vs DMSO	0.8747	4	
	2.5 vs DMSO	0.8747	4	
	5 vs DMSO	0.7323	4	
	10 vs DMSO	0.0001	4	b

Nebivolol hydrochloride	DMSO		3	
	0.5 vs DMSO	0.8926	4	
	1 vs DMSO	0.8719	4	
	2.5 vs DMSO	0.7454	4	
	5 vs DMSO	0.4518	4	
	10 vs DMSO	0.0495	4	a
Nilotinib	DMSO		3	
	0.5 vs DMSO	0.0391	3	a
	1 vs DMSO	0.0242	3	a
	2.5 vs DMSO	0.0190	3	a
	5 vs DMSO	0.0103	3	a
	10 vs DMSO	0.0132	3	a
	20 vs DMSO	0.0038	3	b
Pazopanib hydrochloride	DMSO		3	
	0.5 vs DMSO	0.6561	3	
	1 vs DMSO	0.7353	3	
	2.5 vs DMSO	0.6968	3	
	5 vs DMSO	0.0190	3	a
	10 vs DMSO	<0.0001	3	b
Pimecrolimus	DMSO		3	
	0.5 vs DMSO	0.8672	3	
	1 vs DMSO	0.7898	3	
	2.5 vs DMSO	0.1010	3	
	5 vs DMSO	0.0191	3	a
	10 vs DMSO	<0.0001	3	b
	20 vs DMSO	<0.0001	3	b
Podofilox	DMSO		3	
	0.5 vs DMSO	0.0059	3	b
	1 vs DMSO	0.0116	3	a
	2.5 vs DMSO	0.0019	3	b
	5 vs DMSO	0.0010	3	b
	10 vs DMSO	0.0005	3	b
	20 vs DMSO	0.0003	3	b
Rabeprazole sodium	DMSO		3	
	0.5 vs DMSO	0.9094	3	
	1 vs DMSO	0.9619	3	
	2.5 vs DMSO	0.9739	3	
	5 vs DMSO	0.9018	3	
	10 vs DMSO	0.0013	3	b
Tazarotene	DMSO		3	
	0.5 vs DMSO	0.0476	3	a
	1 vs DMSO	0.0160	3	a
	2.5 vs DMSO	0.0097	3	b
	5 vs DMSO	0.0015	3	b
	10 vs DMSO	0.0002	3	b
	20 vs DMSO	<0.0001	3	b
Thiotepa	DMSO		3	
	0.5 vs DMSO	0.5433	3	
	1 vs DMSO	0.3125	3	
	2.5 vs DMSO	0.1575	3	
	5 vs DMSO	0.0328	3	a
	10 vs DMSO	0.0328	3	a
	20 vs DMSO	0.0066	3	b
Tretinoin	DMSO		3	
	0.5 vs DMSO	0.0027	3	b
	1 vs DMSO	<0.0001	3	b
	2.5 vs DMSO	<0.0001	3	b
	5 vs DMSO	<0.0001	3	b
	10 vs DMSO	<0.0001	3	b
	20 vs DMSO	<0.0001	3	b

Appendix Figure S2	Dunnett's multiple comparison test	P value	N	Label
Cholesterol	DMSO		9	
	1 vs DMSO	0.5056	3	
	10 vs DMSO	0.3227	3	
Pregnenolone	DMSO		9	
	1 vs DMSO	0.7686	3	
	10 vs DMSO	0.9728	3	
Progesterone	DMSO		9	
	1 vs DMSO	0.0082	3	b
	10 vs DMSO	0.005	3	b
Deoxycorticosterone	DMSO		9	
	1 vs DMSO	0.8273	3	
	10 vs DMSO	0.0003	3	b
Corticosterone	DMSO		9	
	1 vs DMSO	0.7445	3	
	10 vs DMSO	0.8596	3	
Aldosterone	DMSO		9	
	1 vs DMSO	0.9228	3	
	10 vs DMSO	0.9727	3	
17alpha-hydroxypregnenolone	DMSO		9	
	1 vs DMSO	0.1796	3	
	10 vs DMSO	0.1796	3	
17alpha-hydroxyprogesterone	DMSO		9	
	1 vs DMSO	0.4824	3	
	10 vs DMSO	0.0001	3	b
11-deoxycortisol	DMSO		9	
	1 vs DMSO	0.0769	3	
	10 vs DMSO	0.0002	3	b
Cortisol	DMSO		9	
	1 vs DMSO	0.7647	3	
	10 vs DMSO	0.9961	3	
DHEA sulfate	DMSO		9	
	1 vs DMSO	0.3937	3	
	10 vs DMSO	0.1225	3	
DHEA	DMSO		9	
	1 vs DMSO	0.9478	3	
	10 vs DMSO	0.0002	3	b
Androstenedione	DMSO		9	
	1 vs DMSO	0.2769	3	
	10 vs DMSO	0.7204	3	
Estrone	DMSO		9	
	1 vs DMSO	0.9988	3	
	10 vs DMSO	0.5123	3	
Estriol	DMSO		9	
	1 vs DMSO	0.6882	3	
	10 vs DMSO	0.8248	3	
Testosterone	DMSO		9	
	1 vs DMSO	0.0099	3	
	10 vs DMSO	0.0001	3	
Estradiol	DMSO		9	
	1 vs DMSO	0.9647	3	
	10 vs DMSO	0.6144	3	
Dihydrotestosterone	DMSO		9	
	1 vs DMSO	0.6923	3	
	10 vs DMSO	0.9496	3	

Appendix Figure S3	Welch's t-test	P value (two-tailed)	N	Label
	Control		3	
	ABL1 vs Control	<0.0001	3	**
	Control		3	
	ABL2 vs Control	0.0060	3	**
	Control		3	
	BCR vs Control	0.0039	3	**
	Control		3	
	SRC vs Control	<0.0001	3	**

Appendix Figure S5A	two-way ANOVA	P value	N	Label
	Interaction	0.4416		
	Row Factor	<0.0001		**
	Column Factor	<0.0001		**
	Sidak's multiple comparisons test			
	DMSO:0.1 mM Luc vs DMSO:0.2 mM Luc	0.0085	6	**
	DMSO:0.1 mM Luc vs 20 μM DHEA:0.1 mM Luc	<0.0001	6	**
	DMSO:0.1 mM Luc vs 20 μM DHEA:0.2 mM Luc	<0.0001	6	**
	DMSO:0.2 mM Luc vs 20 μM DHEA:0.1 mM Luc	<0.0001	6	**
	DMSO:0.2 mM Luc vs 20 μM DHEA:0.2 mM Luc	<0.0001	6	**
	20 μM DHEA:0.1 mM Luc vs 20 μM DHEA:0.2 mM Luc	0.0006	6	**

Appendix Figure S5B	two-way ANOVA	P value	N	Label
	Interaction	0.0740		
	Row Factor	<0.0001		**
	Column Factor	<0.0001		**
	Sidak's multiple comparisons test			
	DMSO:pulsed vs DMSO:tonic	<0.0001	6	**
	DMSO:pulsed vs 20 μM DHEA:pulsed	<0.0001	6	**
	DMSO:pulsed vs 20 μM DHEA:tonic	<0.0001	6	**
	DMSO:tonic vs 20 μM DHEA:pulsed	0.3664	6	
	DMSO:tonic vs 20 μM DHEA:tonic	<0.0001	6	**
	20 μM DHEA:pulsed vs 20 μM DHEA:tonic	<0.0001	6	**

Appendix Figure S5C	two-way ANOVA	P value	N	Label
	Interaction	<0.0001	6	**
	Row Factor	0.0012	6	**
	Column Factor	<0.0001	6	**
	Sidak's multiple comparisons test			
	insulin (+):DMSO vs insulin (+):Dasatinib	<0.0001	6	**
	insulin (+):DMSO vs insulin (+):Nilotinib	<0.0001	6	**
	insulin (+):DMSO vs insulin (-):DMSO	0.0228	6	*
	insulin (+):DMSO vs insulin (-):Dasatinib	<0.0001	6	**
	insulin (+):DMSO vs insulin (-):Nilotinib	0.0001	6	**
	insulin (+):Dasatinib vs insulin (+):Nilotinib	<0.0001	6	**
	insulin (+):Dasatinib vs insulin (-):DMSO	<0.0001	6	**
	insulin (+):Dasatinib vs insulin (-):Dasatinib	0.1527	6	
	insulin (+):Dasatinib vs insulin (-):Nilotinib	<0.0001	6	**
	insulin (+):Nilotinib vs insulin (-):DMSO	<0.0001	6	**
	insulin (+):Nilotinib vs insulin (-):Dasatinib	<0.0001	6	**
	insulin (+):Nilotinib vs insulin (-):Nilotinib	0.0001	6	**
	insulin (-):DMSO vs insulin (-):Dasatinib	<0.0001	6	**
	insulin (-):DMSO vs insulin (-):Nilotinib	<0.0001	6	**
	insulin (-):Dasatinib vs insulin (-):Nilotinib	<0.0001	6	**
