# Supplementary material for: Identification of circadian clock modulators from existing drugs
Source: EMBO Mol Med. 2018 Apr 17;10(5):e8724. doi: 10.15252/emmm.201708724 (PMC5938619; doi:10.15252/emmm.201708724)
Supplement: Supplementary file 2 — Expanded View Figures PDF [file EMMM-10-e8724-s002.pdf]

Expanded View Figures

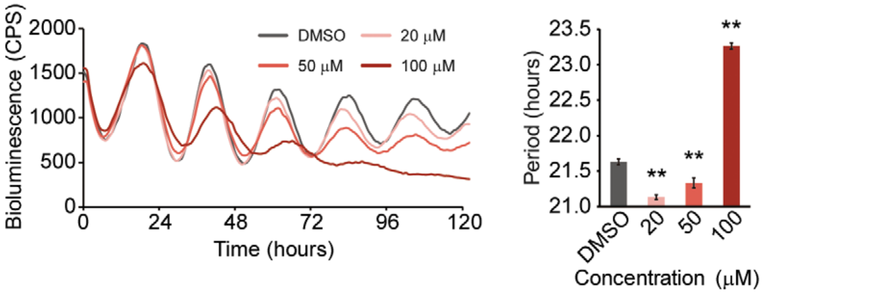

Figure EV1. High doses of DHEA lengthen circadian period.

U2OS cells were treated with 0, 20, 50, and 100 μM DHEA in 0.2% DMSO. Histograms show effects on circadian period, and results are presented as the mean ± SEM (*n* = 6). Data were analyzed by one-way ANOVA, followed by a Dunnett's test (***P* < 0.01). All statistical information is shown in Appendix Table S2.

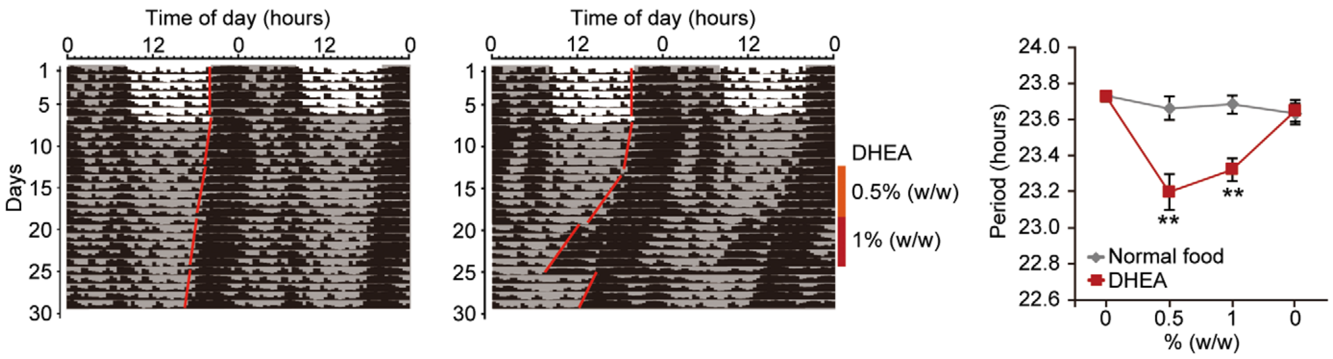

Figure EV2. Effect of DHEA on circadian rhythms in body temperature.

Representative double-plotted temperature records from control (*n* = 8; left) and DHEA-treated (*n* = 13; middle) animals. Free-running period (mean ± SEM) was determined (right) and analyzed by two-way ANOVA, followed by a Sidak's multiple comparisons test (***P* < 0.01). All statistical information is shown in Appendix Table S2.

© 2018 The Authors

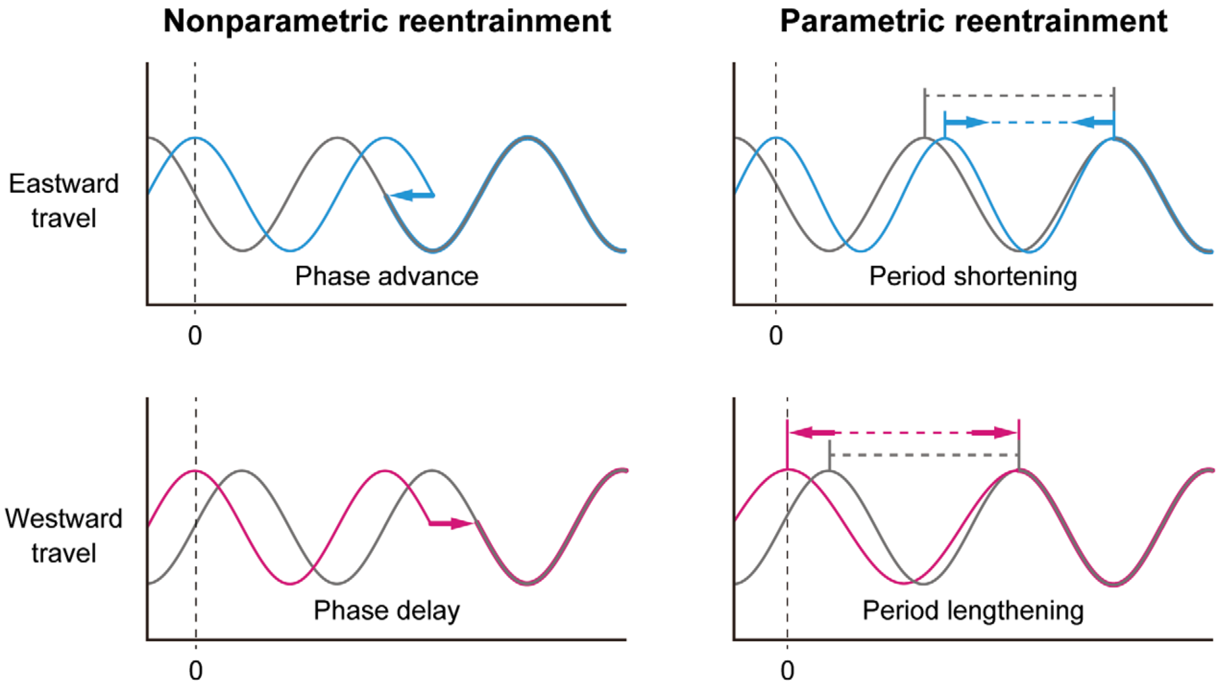

Figure EV3. Possible mechanisms of overcoming jet-lag.

Two models have been proposed to explain the mechanisms by which circadian clocks are entrained to environmental LD cycles. In the "nonparametric model", entrainment is achieved by a phase shift caused by environmental stimuli. In contrast, the "parametric model" focuses on the angular velocity or rate of motion of the circadian clock, and entrainment is achieved by modulation of the free-running period (Daan & Aschof, 2001). Therefore, compounds that increase the magnitude of the phase shift are predicted to reduce jet-lag through a nonparametric mechanism, whereas period-shortening and period-lengthening compounds (i.e., compounds that affect angular velocity) might reduce jet-lag either by accelerating or slowing down the clock, thus accelerating re-entrainment to travel eastward or westward, respectively.

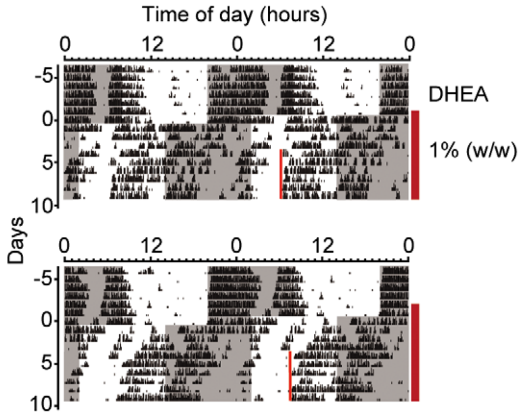

Figure EV4. Chronic DHEA leads to a stable phase advance in activity onset in some mice.

Double-plotted actograms of two DHEA-treated mice from the experiment shown in Fig 3A. DHEA was administered in powdered food (1.0% w/w; vertical line on the right) during re-entrainment to a 6-h phase-advanced LD cycle. These results show that, in some mice, chronic DHEA treatment leads to a relatively stable phase advance in activity onset ~4–6 h prior to lights off.

 © 2018 The Authors

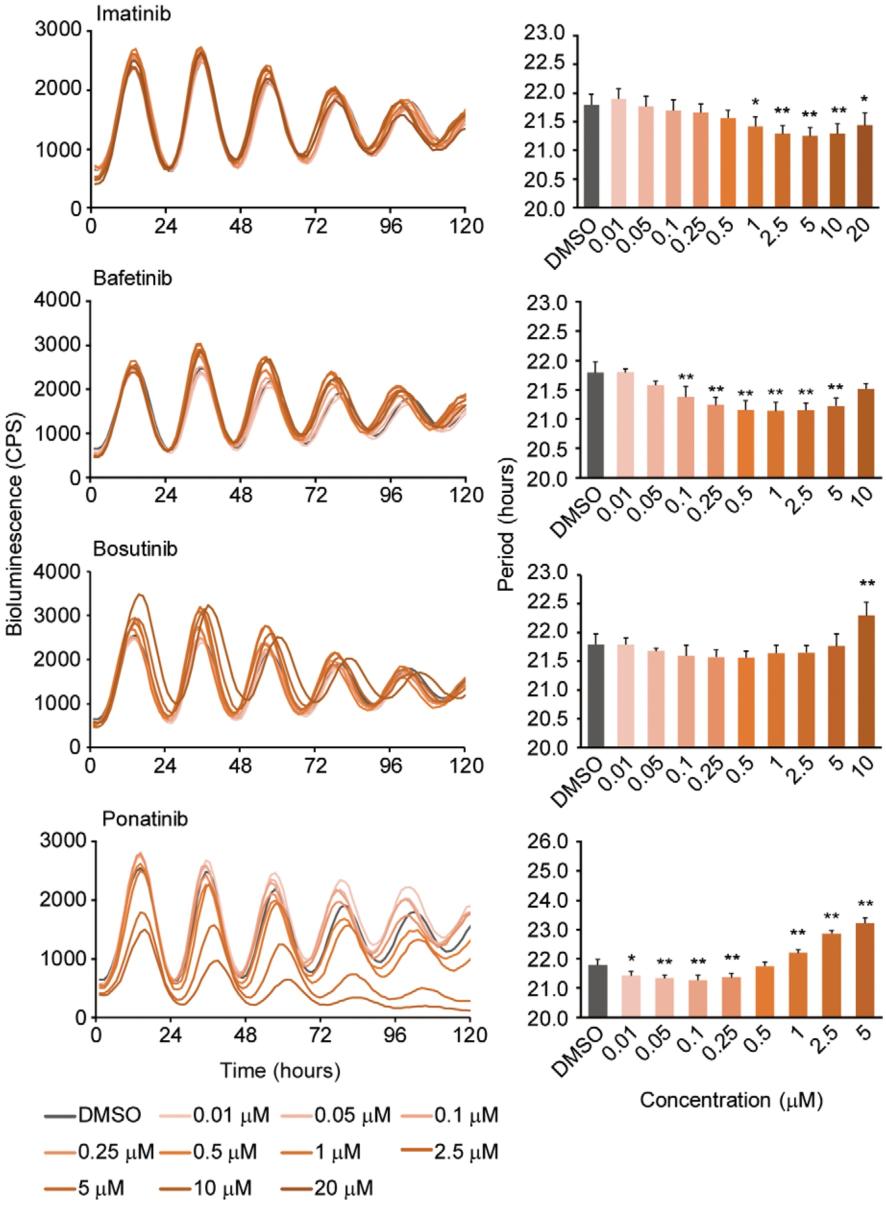

Figure EV5. Dose-dependent effects of BCR-ABL tyrosine kinase inhibitors on circadian period.

Luminescent traces from one of three or four independent experiments are shown. Circadian period was determined by curve fitting. Data are the mean ± SEM of three or four independent experiments and were analyzed by one-way ANOVA, followed by a Dunnett's test (*$P < 0.05$, **$P < 0.01$). All statistical information is shown in Appendix Table S2.

© 2018 The Authors
